# Determinants of WASH programmes adoption in flood-prone Tsholotsho District, Zimbabwe

**DOI:** 10.4102/jamba.v16i2.1803

**Published:** 2024-10-22

**Authors:** Mlamuleli Tshuma, Johannes A. Belle, Alice Ncube

**Affiliations:** 1 Disaster Management Training and Education Centre, Faculty of Natural and Agriculture Sciences, University of the Free State, Bloemfontein, South Africa; 2 Department of Development Studies, Faculty of Humanities and Social Sciences, Lupane State University, Bulawayo, Zimbabwe

**Keywords:** water, sanitation and hygiene, resilience, flood hazard, probit regression, logit regression

## Abstract

There is an increased frequency of floods in different parts of the world due to Climate Change and many countries in Sub-Saharan Africa face WASH challenges which have worsened during flood and drought incidences. Tsholotsho District has been experiencing an increased frequency of floods over the years and WASH situation has continuously deteriorated. To build the resilience of communities, there are so many programmes that have been introduced toaddress WASH challenges. The paper aimed at assessing the various determinants influencing the adoption of WASH programmes in flood prone Tsholotsho District. A quantitative approach was used to collect data from Household heads in wards 5, 6, 7, and 8 using Questionnaires. A total of 218 Questionnaires were administered in all four wards. A Probit regression analysis and Zero-inflated ordered logit regression analysis were then done using Statistical Package for Social Sciences (SPSS). This study revealed that the coefficient of access to treated water, gender, source of water, level of education, and marital status is positive and statistically significant with the adoption of WASH programmes by the household head in the study area. These factors were also revealed to influence the level of adoption of WASH programmes.

**Contribution:**

There is a positive and significant relationship between access to safe water, source of water, level of education, gender, age and marital status and WASH programmes. Therefore, there is a need to consider the determinants of the adoption of WASH programmes to effectively build the resilience of communities.

## Introduction

Since the start of the new millennium, notable strides have been made to improve access to water, sanitation and hygiene (WASH) services; however, to date, 709 million people in sub-Saharan Africa (SSA) still lack access to basic sanitation services (Ohwo & Agusomu 2019). Rapid population growth and rapid urbanisation in SSA pose major development problems such as limited access to improved water and sanitation services (Mackinnon et al. 2019). Sustainable development goal number 6, which aims to achieve universal and equitable access to safe, affordable drinking water as well as sanitation and hygiene for all by 2030 remains a pipe dream for developing countries with only 7 years left. Inadequate access to improved WASH services has varied and complex health consequences for people mostly living in rural areas (Momberg et al. 2021). It causes a serious burden of diarrhoeal diseases and remains the major cause of child mortality around the globe (Masangkay et al. 2020). Neglected tropical diseases (NTD) are still high in SSA owing to a lack of access to improved WASH services. Sub-Saharan Africa has the lowest levels of access to improved WASH services especially in rural communities (Munamati, Nhapi & Misi 2016). In urban areas, there has been increased pressure on WASH facilities because of rapid urbanisation necessitated by climate change, which is forcing people to migrate to urban areas for alternative livelihood sources (Quattrochi et al. 2021).

About 63% of the SSA population lives in rural areas and only 28% of this population has access to basic water and sanitation services (Momberg et al. 2017). The 10 worst countries in terms of water and sanitation coverage are found in SSA (Rivero, Morais & De Sousa Pereira 2020). The progress of WASH coverage in rural areas is worse than in urban areas despite the fact that there is a higher number of non-governmental organisations (NGOs) implementing WASH programmes in rural areas than in urban areas. Chikadaya, Madziyire and Munjanja (2018) argued that a reduction in open defecation does not necessarily mean an improvement in sanitation coverage as some households use sanitation facilities like traditional uncovered pits, buckets and hanging toilets leading to biological contamination of groundwater causing severe health impacts (Fuente et al. 2020). Poor communities are perceived to lack basic WASH services because their socio-economic status is a critical determinant of access to WASH services.

Despite significant efforts to develop rural infrastructure, the imbalance between urban and rural services remains a distinctive feature of the sector in Zimbabwe today (Madzingamiria, Schouten & Blokland 2015). About 98% of those without an improved drinking water source live in rural areas and up to 42% of the rural population practises open defecation (World Health Organization [WHO] 2020). Hidden behind the coverage statistics, there has also been a significant decline in the quality of urban and rural services which include poorer water quality, intermittent supplies and longer walking distances (Ndlovu & Bhala 2016). Sanitation coverage has stagnated since 1990, with only a slow reduction in open defecation (Ngwenya et al. 2018). Faecally transmitted infections are high in flood-prone areas as WASH facilities during flooding are affected (Karthe et al. 2017). Although there are numerous WASH programmes rolled out after a flood, both governmental and NGO driven, these communities are still affected by the impacts of WASH problems.

## Background information on water, sanitation and hygiene situation in Tsholotsho

Tsholotsho district, which is in Matabeleland North province of Zimbabwe, is one of the rural districts that have a high frequency of WASH-related hazards, especially in its flood-prone areas. According to Tsholotsho District Hospital (2018) records, there were 486 cases of dysentery, 257 cases of scabies, 72 cases of bilharzia and 6569 cases of diarrhoea in the 2017 and 2018 rainfall seasons. Most of these cases are recorded in the flood-prone wards, which are wards 5, 6, 7 and 8. Most of the households in all the 22 wards in the Tsholotsho district still use the pit latrine system, cat system and open defecation system. This has, over the years, been posing a threat of sanitation-related illnesses around the district. Because of a lack of improved access to safe drinking water, some of the recommended hygiene performances like hand washing under running water are not being practised in some areas resulting in increased exposure to WASH-related hazards, especially the current coronavirus disease 2019 (COVID-19) pandemic (Dzinamarira & Musuka 2021).

Most diarrhoeal deaths among children below the age of 5 are related to poor WASH facilities in the Tsholotsho district (Bastaraud et al. 2020). The diarrhoeal diseases also affect children’s dietary status with related health and socio-economic concerns (McQuade et al. 2020). Usage of unsafe drinking water in food preparation is also an explanation for a substantial amount of diarrhoeal illnesses among newly born and young children in flood-prone wards of Tsholotsho (Humphrey et al. 2019). As such, 25% of stunted growth cases in young children, below the age of 2 years, are also associated with five or more diarrhoeal incidents per child per year (Mavhura, Manyena & Collins 2017). Lengthy contact with faecal pathogens likewise increases the chances of environmental enteric dysfunction (EED) in children in flood-prone wards of Tsholotsho where people drink water from unprotected water sources (Case & Mwinyi 2008). This is mainly an asymptomatic condition which can cause chronic swelling, low nutrient absorption of the intestine and debilitated barrier purpose of the small intestines (WHO 2018).

**FIGURE 1:**
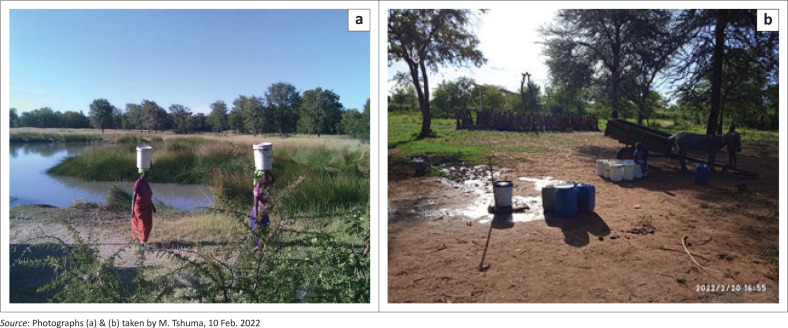
(a) Women carrying water from a pond and (b) piped water scheme water point.

In the Tsholotsho District, water supply systems frequently have extended downtime, high breakdown incidences and insufficient and untrustworthy supplies (Madzingamiria et al. 2015). Because of the low water table in the area and the type of soil, there are several water points that collapsed and were abandoned, yet communities desperately need them. During flooding, some boreholes have collapsed making communities resort to insecure water sources. Poor access to safe water and sanitation, as well as a lack of hygiene have profound impacts on women and girls (SNV 2017). These impacts include adverse pregnancy outcomes, maternal mortality, menstrual hygiene management problems, as well as violence and psychosocial stress (WHO 2017a). The lack of access to safe water forces women to walk for long distances carrying buckets or pushing wheelbarrows in search of water causing spinal damage, hernias, genital prolapse and amplified risk of impulsive abortion (Telles, Reddy & Nagendra 2019) (Figure 1). Limited access to safe water and sanitation and poor hygiene practices increase risks for the development of urogenital contagions to women and adolescent girls, of which some cases were reported in Tsholotsho district (Neseni & Guzha 2009).

In the Tsholotsho district, NTDs, which are viral, as well as parasitic and bacterial diseases are mostly linked to poor WASH services and are prevalent in flood-prone wards (Dube, Mtapuri & Matunhu 2018b). Ineffective water management boosts the breeding of mosquitoes and related spread of malaria and dengue, which pose great risks to pregnant women (Hove et al. 2019). Deprived sanitation upsurges the risk of soil transmitted helminth (STH) infections which result in anaemia and listeria, and increase the danger of maternal fatality and pre-term birth (Dube, Mtapuri & Matunhu 2018a). The bulk of the illness problems related to STH are found mostly in children of school-going age (UNICEF 2012). These infections can lead to undernutrition and growth stunting as well as impaired cognitive development.

Poor WASH practices and facilities cause chronic worm infections such as whipworm (*Trichuris trichiura*) infections and roundworm (*Ascaris lumbricoides*) infections in children who do not wear shoes (WHO & UNICEF 2019). Drinking dirty water causes hookworm (*Necator americanus* and *Ancylostoma duodenale*) (Momberg et al. 2021). Trachoma which is the biggest cause of blindness is also linked to poor WASH services (Kearns et al. 2019). Schistosomiasis resulting from drinking contaminated water results in long-lasting and often irreparable liver and kidney failure and children are mostly affected (Harvey & Reed 2004).

According to Rural Water Information Management Systems (RWIMS 2018), there are 1117 boreholes in Tsholotsho, of which 709 are functional boreholes, 209 are non-functional because of mechanical reasons and 42 boreholes have dried up. Meanwhile, 157 boreholes have been abandoned either because they produce salty water, or they failed to yield any water according to the District Development Fund (DDF). Areas like ward 7 (Patalika) have boreholes with salty water, and communities rely on scattered ponds around the ward. There are four piped water systems in the district which serve both the communities and institutions but have several leaks along their lines and some have become non-functional. There is limited community participation in the development process, ownership and overall responsibility of water supply facilities for their effective operation and maintenance (Dube et al. 2018b). In most cases, a top-down approach in all decision-making has created sustainability challenges for water supply facilities (Frischen et al. 2020). The efforts to improve WASH in most rural areas are marred by a lack of accountability and financial resources, corruption, inefficient management and low prioritisation of funding (WHO 2020). Currently, there is a higher inherent risk when policies are made by authorities and do not include the communities for their input (Displacement Report, Tsholotsho District 2018). Thus water, sanitation and hygiene problems in flood-prone areas in Tsholotsho District indicate that there is a lack of community participation.

Communities in Tsholotsho mainly get their water from boreholes and open water sources. Thebe and Maviza (2019) revealed that the water availability situation is mainly in two phases; that is, during the period December–June, water will be available and from July–November, water will be scarce. During the months when water is scarce, there will be competition between humans and livestock, all relying on the few functional boreholes and earth dams. In ward 7, most parts such as Mpilo and Pelandaba water are not safe for drinking especially during the winter season as a result of high salinity. The water is scarce in the period June–November as most of the boreholes will be dry. During the rainfall season, most communities get their water from open sources, as the closest working boreholes are too far to make it a feasible option. Tsholotsho District in Zimbabwe has, over the years, experienced an increased frequency of flooding, which has also exacerbated the WASH challenges for the communities. Therefore, the main aim of this article is to unravel the determinants of adoption and level of adoption of WASH programmes in flood-prone communities in Tsholotsho District.

## Research methods and design

A quantitative approach was used to collect data from household heads in wards 5, 6, 7 and 8 using questionnaires. The flood-prone wards were purposively selected, and these are ward (Sipepa) 5 with 1026 households, ward (Jimila) 6 with 1400 households, ward 7 (Patalika) with 860 households and ward 8 (Mbiriya) with 1120 households. This study used the Raosoft software package to calculate the sample size from the study population using equation 1:

x=Z(c100)2r(100−r)n=Nx((N−1)E2+x)E=Sqrt[(N−n)xn(N−1)] [Eqn 1]

Using the formula, sample size ***n*** and error margin as ***E*** are provided where ***N*** represents the population size, ***r*** is the fraction of responses that the researcher is concerned about and *Z*(*c*/100) is the critical value for the confidence level ***c*** (Ivankova & Wingo 2018). Using the Raosoft online sample calculator, the total sample for the questionnaire survey generated was 239 respondents for all four wards. A total of 239 questionnaires were administered pro rata to the total number of households in each of the four wards using simple random sampling. The collected data was then analysed using Statistical Package for Social Sciences (SPSS). A probit regression analysis was conducted to determine the factors influencing the adoption of WASH programmes, along with a zero-inflated ordered logit regression analysis to examine the factors affecting the level of adoption of these programmes.

### Ethical considerations

Ethical clearance to conduct the study was obtained from the University of the Free State, General/Human Research Ethics Committee (GHREC) (No. UFS-HSD2021/1858/21).

## Results and discussions

### Water, sanitation and hygiene situation in Tsholotsho District

In all four wards, 62.4% of the respondents revealed that they rely on community boreholes, with 19.7% of the respondents depending on unprotected wells. The percentage of respondents relying on an unprotected well is a cause for concern because this water source can be a source of waterborne diseases, especially if water is used without treating it. It is worth noting that there are respondents who also had private taps in their homesteads and these were 4.6% of the total respondents. There were few respondents who had a private borehole and those who relied on a protected well with 0.9% and 0.5% respectively. All the respondents in ward 8 indicated that they relied on a community borehole. In ward 7, 95.6% of the respondents relied on unprotected wells. Table 1 shows sources of water that service all four wards and the percentage of the respondents who rely on these sources.

**TABLE 1:** Sources of water for Tsholotsho communities.

Name of ward	Source of water (%)					
	Private tap	Private borehole	Protected well	Community tap	Community borehole	Unprotected well
5	8.7	-	-	26.1	65.2	-
6	7.4	2.9	-	20.6	69.1	-
7	2.2	-	2.2	-	-	95.6
8	-	-	-	-	100.0	-

**Total**	**4.6**	**0.9**	**0.5**	**11.9**	**62.4**	**19.7**

The quality of water in all four wards was classified into three main categories: very good, good and poor. The quality of water was very good in wards 5, 6 and 8 with 71.7%, 67.6% and 100%, respectively. About 19.1% of respondents in ward 6 indicated that water quality was poor. In ward 7, about 95.6% of the respondents classified water quality in their ward as poor. This is because of the fact that water in ward 7 is too saline and in some places, humans are not able to drink it. A larger number of respondents summing to 64.5% indicated that they did not treat their water. This may be because of a lack of awareness and level of education of the respondents on WASH issues that are of concern in their areas.

In all four wards, a total of 64.5% of respondents indicated that they do not treat water before using it. Only about 35.5% of the respondents revealed that they treat their drinking water with 89.9% of these respondents boiling water before use. About 6.3% of the respondents relied on water treated with water guard or chlorine. These were mainly from ward 6, where the flood victims of the 2016–2017 rainfall season were relocated (Table 2).

**TABLE 2:** Sanitation facilities in Tsholotsho communities.

Name of ward	Which sanitation facility do you have (%)			
	Pit latrine	Flush toilet	Open space	Other
5	17.4	21.7	60.9	-
6	67.6	4.4	27.9	-
7	24.4	11.1	55.6	8.9
8	88.1	-	-	11.9

**Total**	**53.7**	**8.3**	**33.0**	**5.0**

There were very few respondents who filtered water with a cloth before using it or those who allowed water to settle before using it with 2.5% and 1.3% of the respondents, respectively. The percentage of respondents who indicated that they do not treat water corresponds with the survey by SNV (2019) which revealed that 70% of the people in rural Zimbabwe rely on unsafe drinking water sources and do not treat water before use. This also coincides with the UN (2020) report which revealed that diarrhoeal diseases related to water and sanitation are the principal killers of infants in sub-Saharan Africa where most people, especially in rural areas still depend on unsafe water sources.

### Probit regression analysis of determinants of adoption of water, sanitation and hygiene programmes

This section presents the analysis of the findings of the study based on the results of the probit regression analysis. Probit regression analysis is used in this study to estimate the factors that influence the adoption of WASH programmes by communities. These WASH programmes are mainly offered by the government through DDF, Rural District Council (RDC), NGOs, Faith Based Organisations (FBOs) and the pivate sector. The result of factors that influence the adoption of WASH programmes in Tsholotsho District particularly in areas that are prone to flooding which was modelled with probit regression is presented in Table 3.

**TABLE 3:** Probit regression model of determinants of adoption of water, sanitation and hygiene programmes.

Determinants	Coefficient	s.e.	*P*	dy/dx	s.e.	*P*
Gender	−0.124	0.210	0.554	−0.043	0.072	0.553
Treated water	0.364	0.219	0.096*	0.126	0.074	0.091*
Source of water	0.249	0.092	0.007***	0.086	0.030	0.005***
Age	0.001	0.005	0.906	0.000	0.002	0.906
Distance to nearest water source	−0.024	0.074	0.741	−0.008	0.025	0.741
Level of education	−0.076	0.118	0.521	−0.026	0.041	0.519
Head of household	0.005	0.102	0.964	0.002	0.035	0.964
Rural District Council	−0.225	0.195	0.250	−0.078	0.067	0.246
District Development Fund	0.572	0.134	0.000***	0.198	0.040	0.000***
NGOs	0.196	0.084	0.019**	0.068	0.028	0.015**
Access to other income	−0.010	0.220	0.966	−0.003	0.076	0.966
Access to health facility	−0.065	0.230	0.778	−0.022	0.080	0.778
Constant	2.337	0.979	0.017*	-	-	-
Pseudo *r*-squared	0.127	-	-	-	-	-
Chi-square	37.393	-	-	-	-	-
Akaike crit. (AIC)	284.332	-	-	-	-	-
Bayesian crit. (BIC)	331.324	-	-	-	-	-
Prob > Chi^2^	0.000	-	-	-	-	-

NGO, non-governmental organisations; s.e., standard error; AIC, akaike information criterion; BIC, Bayesian information criterion.

*** *p* < 0.01,

** *p* < 0.05,

* *p* < 0.1.

The coefficient of access to treated water is positive and statistically significant with the adoption of WASH programmes by the household head in the study area. This implies that the adoption of WASH increases with increased access to treated water. The marginal analysis shows that a unit increase in access to treated water increases the adoption of WASH programmes by 13%. This is in alignment with the findings documented in the WHO (2015) report which posited that access to safe drinking-WASH services is a fundamental element of healthy communities and has an important positive impact on nutrition. The result is also in tandem with the study of Ahmed et al. (2022) who also found a positive and significant relationship between access to treated water and WASH programmes in their study on the impact of WASH-related interventions and policy on children’s school performance in Pakistan.

Another positive significant coefficient which increases the adoption of WASH programmes is the access to NGO programmes. This indicates that the more people have access to NGO programmes, the higher the rate of adoption of WASH programmes. The analysis points out that an increase in NGO programmes has a positive influence on the adoption of WASH programmes by the communities. This gives affirmation to a study by Etongo et al. (2018) in Uganda which asserted that there are many NGOs in sub-Saharan Africa funding and implementing WASH programmes. The study also reveals that unlike Local and National Governments, RDCs and FBOs, NGOs have budgetary support for their programmes, hence their projects have achievable timelines and they also support the operation and maintenance of water and sanitation infrastructure.

Furthermore, the coefficient of access to an improved water source and improved sanitation facility is also positive and significantly improves the adoption of WASH programmes by the communities. An increase in access to improved water sources and sanitation facilities also means an increase in the adoption of WASH programmes by a marginal of 8%. The inference drawn from this is that the adoption of WASH programmes increases with increased access to an improved water source and sanitation facility. Therefore in Tsholotsho District when there are many piped water schemes for the communities, more WASH programmes are adopted by the communities. This result validates a study by Joshi (2011) in Western Nepal which revealed that access to improved water and sanitation facilities significantly gives communities full control of the project cycle enhancing behaviour change which is an indication of the adoption of a WASH programme. A study by Ohwo and Agusomu (2019) in sub-Saharan Africa also confirms the result of this probit regression analysis as it shows that the overall improved water and sanitation interventions are more likely to promote collective action by communities ensuring sustainability of the projects.

It is worth noting that there are variables that had no significant impact on the adoption of WASH programmes and these included gender, distance to the nearest water source, level of education, source of income and access to health. Statistically, this implies that these variables had a negative influence on the adoption of WASH programmes. However, of interest to this study were the variables which statistically had a positive and significant influence on the adoption of WASH programmes.

### Zero-inflated ordered logit regression model of factors influencing the level of adoption of water, sanitation and hygiene programmes

In order to determine the factors that influence the adoption of WASH programmes in Tsholotsho District, the zero-inflated ordered logit regression model was used. The results of the analysis were captured and presented in Table 4.

**TABLE 4:** Zero-inflated ordered logit regression model of factors influencing the level of adoption of water, sanitation and hygiene programmes.

Factors	Coefficient	s.e.	*P*
Marital status	0.374	0.406	0.357
Gender	0.489	0.441	0.268
Treated water	1.430	0.890	0.108
Source of water	1.226	0.617	0.047**
Distance to nearest water source	0.170	0.114	0.135
Level of education	0.491	0.272	0.071*
Head of household	0.210	0.155	0.175
Rural District Council	1.033	0.615	0.093*
District Development Fund	−2.970	1.352	0.028**
NGOs	1.112	0.495	0.025**
Access to other income	−0.090	0.331	0.787
Inverse mill ratios	18.532	6.908	0.007***
**Inflate**			
Dependency on remittances	−0.933	1105.226	0.999
_cons	15.739	793.288	0.984
/cut1	20.759	9.228	-
/cut2	21.218	9.233	-
/cut3	22.342	9.244	-
/cut4	23.933	9.257	-
Log-likelihood	−253.47089	-	-
Wald Chi^2^(13)	36.25	-	-
Prob > Chi^2^	0.0005	-	-

NGO, non-governmental organisations; s.e., standard error; cons, constant; cut, cut off point.

*** *p* < 0.01,

** *p* < 0.05,

* *p* < 0.1.

The coefficient of 0.210 of household heads’ level of education is positive and statistically significant in influencing the adoption level of WASH programmes. This shows that as the level of education of the household increases, the number of WASH programmes adopted increases. The implication of the result is traceable to the fact that individuals with a higher level of education are expected to have better access to information on quality, safe drinking water and proper hygiene. This is corroborated by the study of DeBruicker Valliant and Winch (2015) who opined that a higher level of education of the household head is positively associated with WASH behaviour practices.

In addition, the coefficient of 1.226 for the source of water is also statistically significant and positively influences the level of adoption of WASH programmes. This implies that the more the improved water resources the higher the number of people in the communities with access to clean and safe drinking water meaning the adoption of WASH programmes increases. A study by Quattrochi et al. (2021) in the Democratic Republic of Congo affirms this finding as it revealed that the gradual increase in the access to and use of safe drinking water has an immense contribution to health productivity and social development of the community. However, the study also lamented the inadequate quality of drinking water which remains a major cause of health problems and poor sanitation in rural areas of the Democratic Republic of Congo. Therefore, the level of adoption of WASH programmes increases as drinking water from improved water sources determines the likelihood of user satisfaction with the quality of water obtained from the source. In Tsholotsho District, there are still struggles of deficiency in water quality and quantity coming out of the facilities, long distance travelled by the households to the nearest water facility as well as costs of construction and maintenance of water facilities.

**FIGURE 2:**
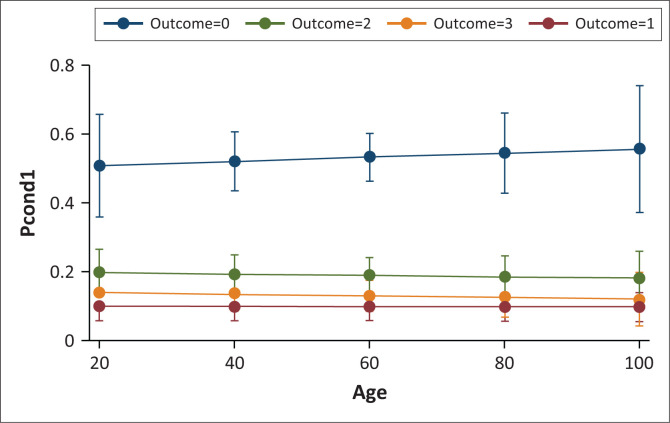
Predictive plot margins.

Being part of an NGO programme also has a positive significance likely to increase the level of adoption of WASH programmes with a coefficient of 1.112. Non-governmental organisations implement many programmes that are related to water and sanitation and the implication is that the more the households that benefit from these programmes means the increase in the level of adoption of WASH programmes. This result of the zero-inflated ordered logit regression is in sync with studies by Kativhu et al. (2018), Sadiqi, Trigunarsyah and Coffey (2017), WHO (2017b) and Lockwood and Le Gouais (2015). These studies show that there are numerous NGOs operating in SSA on water and sanitation programmes as a result of WASH challenges that continue to sicken and kill people as well as affecting the economies. The evidence from these studies shows that the implementation of more NGO programmes and the increase in the number of people accessing those programmes have also increased the uptake of WASH programmes in those particular areas (Figure 2).

Predictive plot margins were used to concentrate on the WASH programme’s adoption rate. The probability of each level of the WASH programme was determined, conditional on ability measured with the age of the household head, by specifying statistic pcond1 which shows variances of adoption levels by each age category. As before, calculations are made at each of the WASH programmes’ four tiers and then plotted using a margins plot. When the yearly age reaches 20, more than half of the households report that they started to embrace the use of WASH programmes, and those that do not embrace them are most likely to use one or two WASH facilities. The likelihood of adoption rises as age does. Being an adopter is more likely to occur as people get older.

The results of this article indicate that there is a need for the drilling of more boreholes across the wards to increase water accessibility, and installing a solarised piped water system would work better for the communities. In order to improve the quality of water, the key informants suggested that there is a need to have systematic periodic water quality tests through the Ministry of Health and Child Care and decommission boreholes that have water quality not meeting the required standard. In areas that have more saline water like parts of wards 7 and 8, there is a need for a piped water scheme where water is channelled from other areas with water that is less saline. There is also a need for continuous and regular community training on issues of WASH management.

## Conclusion

The key determinants of the adoption of the WASH programme in Tsholotsho were discovered to be access to treated water, access to NGO programmes and access to improved water sources. These were factors which either increased or decreased the adoption of WASH programmes at a household level. On the factors influencing the level of adoption, the main findings indicated that the level of education, sources of water and access to NGO programmes were statistically significant and had a positive influence. The findings of this research show that the level of adoption of WASH programmes is still low in Tsholotsho District.

All WASH decisions, projects and programmes should not be imposed on the communities by the national government, local government or development partners. The decisions made at a local level matter in the sustainability of any WASH programme or project to be implemented. Institutional capacities of the government at national and local levels, NGOs and traditional leadership are important for fostering active community participation. Community participation can be improved by formulating WASH bylaws that compel communities to build toilets and punish community members who continue to practise open defecation. Through the use of demand-led sanitation interventions which mainly come from community members, participation of communities can improve.

According to Dube (2020), institutions that are supposed to be helping communities with WASH issues are reeling under difficult economic stress lacking financial and material resources, lacking prioritisation of WASH issues and the zeal to deal with WASH problems effectively which is in agreement with the findings of this study. Rural District Council is obligated to facilitate service delivery within its jurisdiction. Efforts to deal with WASH issues by RDC are affected by financial problems and a lack of prioritisation. The priority in most cases is given to other areas like road rehabilitation instead of WASH issues. The influential councillors in RDC always control decision making which also affects prioritisation of WASH issues.

The national government, local government, NGOs, FBOs, academia and the private sector should conduct more awareness campaigns and encourage and continue educating people on issues around WASH management by supporting communities with the construction of toilets. Widespread community education and awareness can also help in bringing on board community members in any community WASH programme and project. Community participation can be enhanced by formulating WASH bylaws that compel communities to build toilets and impose penalties on members who continue to practise open defecation.
